# Development of a Curcumin Bioadhesive Monolithic Tablet for Treatment of Vaginal Candidiasis

**Published:** 2016

**Authors:** Umme Hani, H.G. Shivakumar, Riyaz Ali M. Osmani, Atul Srivastava, Naga Sravan Kumar Varma

**Affiliations:** *Department of Pharmaceutics, JSS College of Pharmacy, JSS University, SriShivarathreeshwara Nagar, Mysore-570 015, Karnataka, India.*

**Keywords:** Curcumin, Bioadhesion, Vaginal tablets, Candidiasis

## Abstract

The present investigation was designed to formulate a natural tablet for the treatment of vaginal candidiasis in order to eliminate side effects that are caused by existing antifungal drugs. Curcumin has promising antifungal activity in comparison with the existing azole antifungal drugs. Bioadhesive curcumin vaginal tablets were prepared by direct compression with different ratios of biadhesive polymers like xanthan gum, guar gum and HPMC. Curcumin tablets were characterized by studies of friability, hardness, hydration, DSC, mucoadhesion, *In-vitro* release and antifungal activity. DSC and FT-IR data indicate there was no interaction between the drug and the excipients and also polymer concentration has some effects on melting point of curcumin. Formulation F3 showed the best results in terms of swelling and mucoadhesion together with prolonged drug release. The antifungal activity of the Curcumin tablet has demonstrated a significant effect against *Candida albicans*. Hence, the study indicates the possible and effective use of curcumin bioadhesive monolithic vaginal tablet for vaginal candidiasis as a promising natural antifungal treatment.

## Introduction

Curcumin is a major pigment of the Curcuma species, commonly used as a yellow coloring and flavoring agent in foods particularly in South Asia (1). The use of curcumin in traditional medicine and as a household remedy for various diseases, including biliary disorders, anorexia, cough, diabetic wounds, hepatic disorders, rheumatism and sinusitis has been well documented (2). Curcumin has also been reported to possess anti-inflammatory, antioxidant, anticarcinogenic, antimutagenic, anticoagulant, antiarthritic, antibacterial, antifungal, antiprotozoal, antiviral, anti- Alzheimer, anti-psoriatic and neuroprotective activities (3). Studies have shown that curcumin is not toxic to humans. Curcumin exerts anti-inflammatory activity by inhibition of a number of different molecules that play an important role in inflammation (4, 5). Although some works have been done on possible medical applications of curcumin, no studies have been carried out for drug development yet. Work has been carried out on estimation of antifungal activity of curcumin against candida and it was found that curcumin was 2.5 fold more potent than fluconazole at inhibiting the adhesion of candida albicans, and curcumin has synergistic antifungal affects with azoles and polyenes (6).

The potential of mucoadhesive polymers was shown in ocular, nasal, vaginal and buccal drug delivery systems leading to a significantly prolonged residence time of sustained release delivery systems on these mucosal membranes. The vagina has been studied as a favorable site for the local and systemic delivery of drugs, specifically for female related conditions (7).

The human vagina is colonized by microbes, and infections occur when the balance is disturbed. Under healthy conditions, vaginal flora is dominated by lactobacilli, which maintain an acidic pH through production of organic acids at times other than menstruation. Disruptions of vaginal pH or lactobacilli may allow potentially pathogenic microorganisms to grow and dominate (8).

Vaginal candidiasis is a common condition and up to 75% of the women suffer at least one episode of this infection during their life time. *Candida albicans* is the most important cause of vaginal candidiasis, accounting for over 80% of the infection (9). Some of the agents used to date for vaginal candidiasis are clotrimazole, metronidazole, ketoconazole, fluconazole, itraconazole, secnidazole etc but they have side effects. 

Effective bioadhesion to the vagina resulting in a controlled release of the drug depends on bioadhesive polymers used in the formulation. From the literature it was found that HPMC and HPC are the best bioadhesive polymers for vaginal tablets. HPMC is a semisynthetic cellulose derivative and has wide spread use which is mainly due to its generally regarded safe (GRAS) status and biodegradable nature. It is compatible with numerous drugs, accommodates a high level of drug loading and can be incorporated to form a matrix tablet prepared either by direct compression or granulation. HPMC can be used to control the release of both water soluble and water insoluble drugs like curcumin. The most important characteristic of HPMC is the high swellability which has a considerable effect on the release kinetics of incorporated drugs. For water insoluble drugs like curcumin the low viscosity grade polymer in combination with water soluble fillers represent the most common formulation approach to generate consistent erosion controlled system (10).

Xanthan gum is a high molecular weight hetero polysaccharide gum produced by a pure culture fermentation of a carbohydrate with the microorganism Xanthomonas Campestris (11). It has higher retarding ability compared to HPMC. Xanthan gum retards *In-vitro* drug release and provides time independent drug release kinetics from xanthan gum matrices which is independent on the ionic strength of the dissolution medium. More recently it has been proven that xanthan gum play a successful role in sustaining release of drugs from hydrophilic matrix formulations (12). Interaction of HPMC and Guar gum has been reported to form a gel on the surface of tablet which aids in minimizing the burst effect of HPMC based systems. 

Combination of HPMC, xanthan gum and guar gum was used as bioadhesive polymer to formulate Curcumin bioadhesive vaginal tablet. Talc was used as a diluent in the formulation. It can be used as diluents in the concentration of 5-30%. It is widely used as a Dissolution retardant in the development of controlled-release products (13).

Monolithic (matrix) systems is a hydrophilic, hydrophobic or inert matrices in which the drug is homogeneously dispersed or dissolved in a soluble or insoluble matrix and it is released by diffusion and erosion from the polymer (14). Vaginal tablets are simple to manufacture and are comparatively inexpensive. Furthermore, the application is convenient and a sustained drug release can be achieved over several hours, if the delivery system does not disintegrate too early.

Considering curcumin as promising lead compound for the design of new antifungal agent capable of inhibiting the adhesion of *candida albicans*, bioadhesive formulations are developed for the treatment of candidiasis. Formulations developed using curcumin can be used as a herbal vaginal treatment for candidiasis free from side effects.

Hence this study was planned to develop and evaluate an herbal bioadhesive monolithic tablet of curcumin free from side effects for the treatment of vaginal candidiasis.

## Experimental

*Materials*

Curcumin was purchased from Ace rasayan Mysore. Hydroxypropylmethylcellulose (HPMC E 15 LV), Xanthan gum (Pure, Food Grade) and Guar Gum (GG Food grade) from Loba Chemie, Mumbai, India, were used as the mucoadhesive polymers for tablet preparation. Magnesium stearate (Loba Chemie, Mumbai, India) was used as a lubricant and talc was used as a diluent.

*Methods*

*Preparation of simulated vaginal fluid (SVF)*

Vaginal fluid originates from a number of different sources. The fluid is mostly transudate from vaginal and cervical cells and also contains vulvar secretions from sebaceous, sweat, bartholin, and skene glands, cervical mucus, endometrial and oviductal fluids, microorganisms and their metabolic products. pH of SVF varies from 4-5. Because of the limited quantity of human vaginal fluid and its rapid degradation once collected from its source, researchers have developed a simulated vaginal fluid (SVF) in order to simulate the overall components of vaginal fluid. SVF was prepared using 900 mL of distilled water contained in a beaker, NaCl (3.51 g), KOH (1.4 g), Ca (OH)_2_ (0.22 g), bovine serum albumin (0.018 g), lactic acid (2.00 g), acetic acid (1.00 g), glycerol (0.16 g), urea (0.4 g) and glucose (5.00 g) were added and stirred well until complete dissolution occurred. The pH of the SVF was then adjusted to 4.5 using 0.1 N HCl, and the final volume was adjusted to 1 L and used as dissolution media (15, 16).

*Preparation of Curcumin bioadhesive monolithic vaginal (CBMV) tablets*

Direct compression method was used to prepare Curcumin bioadhesive monolithic vaginal tablets containing 20% curcumin and weighing 500 mg using a 10-station tablet machine (minipress-1674, Rimek, India) fitted with round, flat-faced 12 mm punches. Composition of the formulations is listed in Table 1. To obtain a homogenous blend prior to the compression, a particle size of less than 160 μm was selected for all components to avoid any fractional segregation and the formulation well mixed with a mortar and pestle. 

**Table 1:** Composition of curcumin bioadhesive vaginal tablets

**Ingredients (mg)**		**Formulation**									
	**F1**		**F2**	**F3**	**F4**	**F5**	**F6**	**F7**	**F8**	**F9**	**F10**
Curcumin		100	100	100	100	100	100	100	100	100	100
HPMC		200	200	200	200	200	200	-	-	-	100
Xanthan gum		100	125	150	-	-	-	150	100	200	100
Guar gum		-	-	-	100	125	150	150	200	100	100
Magnesium stearate		10	10	10	10	10	10	10	10	10	10
Talc		90	65	40	90	65	40	90	90	90	90

*Tablet physical characterization: Average weight, weight variation, thickness and hardness*

The weight variation test of the tablets was carried out as per the guidelines of Indian Pharmacopoeia. Ten CBMV tablets from each batch were weighed in a Sartorius digital balance and the average weight and standard deviation was calculated. The thickness of ten CBMVT was determined using a digital vernier caliper. The average thickness and standard deviation was calculated. Hardness of the tablet is an indication of its strength. It was tested by measuring the force required to break the tablet across the diameter. The force is measured in kg/cm^2^ and the hardness of about 4 kg/cm^2^ was considered to be satisfactory for uncoated tablets. Tablet requires a certain amount of mechanical strength to withstand the shock of handling during its manufacture, packaging, shipping and dispensing. Hardness of the tablet was determined using Erweka hardness taster. The tensile strength of tablets t was calculated using the following equation: Tensile stress (σt) = 2F/ πDt. (F is the crushing force, D and t is the diameter and thickness of the tablet) (17).

*Tablet physical characterization friability*

Friability is the measure of a tablet’s ability to withstand both shock and abrasion without crumbling during manufacturing, packing, shipping of tablets. The weight of 10 CBMV Tablets was taken and placed in Roche friabilator. The device subjects the tablets to the combined effect of shock and abrasion by utilizing a plastic chamber, which revolves at 25 rpm, dropping the tablets a distance of 6 inches with the revolution. The pre-weighed tablet sample was removed after 100 revolutions, dedusted and reweighed. Tablets that loose less than 0.5 to 1 percent in weight are generally considered acceptable.

$\mathrm{Friability}\left(\%\right)=\frac{\mathrm{Initial wt. of 10 tablets}–\mathrm{final wt. of 10 tablets}}{\mathrm{Initial weight of 10 tablets}}\times 100$

*Drug content estimation*

The Drug content of curcumin in the prepared CBMV tablets was determined by UV spectrometry. 10 tablets were finely powdered, quantity of the powder equivalent to 50 mg of curcumin were accurately weighed and transferred to a 100 mL of volumetric flask. Methanol was added and mixed thoroughly and volume was made up with methanol and filtered. 10 mL of resulting solution was diluted to 100 mL with methanol and the absorbance of the resulting solution was measured at 417 nm using UV Visible spectrometer (Shimadzu, 1800, Japan).

*Physicochemical interaction studies:* *Fourier transforms infrared spectroscopy (FT-IR)*

The spectra were recorded for pure drug, polymer and tablet using Fourier transform infrared spectrometry (FTIR, Shimadzu 8400 S, Japan). Samples were prepared in KBr discs using KBr press (Technosearch, Mumbai, India). The scanning wave number range was 600-4000 cm^-1^.

*Physicochemical* *interaction studies: Differential scanning calorimetry*

The compatibility of curcumin-excipient as well as excipient–excipient compatibility after formulating to a tablet and the effect of compression force on thermal profiles of all the components was assessed using differential scanning calorimetry (DSC). DSC scans of the samples were performed, separately and for formulations (F1-F10) using a DSC TA 60 (Shimadzu). The calorimetric measurements were made with an empty cell (high purity alpha alumina discs of Shimadzu Company) as the reference. The scans were taken under nitrogen atmosphere over a temperature range of 25 to 330 °C and at a scan rate of 20 °C/min.

*Swelling studies*

Each tablet was placed into a stainless steel basket with 200 mesh of aperture and weighed (W1). The basket was then placed in a beaker containing 100 mL SVF of pH 4.5, allowing the tablet to swell at 37 ± 1 °C in an orbital shaker (Remi, Mubai, India) for predetermined times (18). These experiments were performed in triplicate and the final data for % of hydration were calculated using the following equation

% of Hydration = [(W2 −W1) / W2] × 100

Where W1 is weight of the dry tablet with basket

W2 is the weight after immersion in SVF for predetermined time intervals (0.5, 1, 2, 4, 8, 12 and 24 h)

*Ex-vivo mucoadhesion time*

*Ex-vivo* mucoadhesion time was determined after application of tablet on fresh cut sheep vaginal mucosa. The cut tissue (3 cm × 3 cm) was fixed in the internal side of a beaker with cyanoacrylate glue. Each tablet side was wetted with 50 µL of SVF and put in contact with the vaginal mucosa surface applying a finger tip force for 20 s. The beaker was filled (100 mL) by using SVF kept at 37 °C (± 1). Mucoadhesive time was monitored until complete detachment occurred (Table 2) (measurements performed in triplicate). 

**Table 2:** Calculated dimensions (weight, thickness and diameter), hardness, tensile strength, friability, drug content, *Ex-vivo* mucoadhesive time and bioadhesive strength of tablets and their standard deviations

**Formulation**				**Parameters**							
	Mean weight (mg±SD)(n=3)		Thickness (mm±SD) (n=3)		Diameter (mm±SD) (n=3)	Hardness (N)(n=3)	Tensile strength (MPa)	Friability (%)	Drug content (%±SD)(n=3)	Ex-vivo mucoadhesive time (min±SD) (n=3)	Ex-vivo bioadhesive strength (g±SD)(n=3)
**F1**	503±0.2		3.6±0.5		12.1±0.02	53.63 ±0.3	0.790±0.02	0.2	98.6±0.5	19.12±1.2	113±1.2
**F2**	503±0.1		3.6±0.7		12.2±0.01	53.93 ±0.6	0.781±0.01	0.2	99.8±0.6	20.22±0.5	135±1.1
**F3**	504±0.4		3.6±0.5		12.2±0.02	53.53 ±0.4	0.775±0.01	0.2	101.2±0.3	28.28±1.6	154±1.2
**F4**	503±0.3		3.6±0.4		12.2±0.01	53.91 ±0.3	0.781±0.02	0.2	98.9±0.9	8.13±1.0	52±2.1
**F5**	502±0.2		3.6±0.4		12.1±0.01	53.83 ±0.2	0.786±0.01	0.2	101.3±0.3	11.24±0.5	64±1.1
**F6**	504±0.2		3.6±0.5		12.2±0.03	53.93 ±0.7	0.781±0.01	0.2	99.9±0.2	13.27±0.5	80±1.1
**F7**	503±0.1		3.2±0.7		12.1±0.01	48.05 ±0.6	0.789±0.02	0.3	99.5±0.3	6.11±1.3	31±1.1
**F8**	504±0.1		3.1±0.6		12.1±0.01	50.99 ±0.3	0.863±0.01	0.3	102.01±0.5	4.21±1.2	25±1.2
**F9**	499±0.2		3.3±0.6		12.2±0.02	51.97 ±0.2	0.821±0.01	0.3	99.6±0.7	8.06±1.1	38±2.2
**F10**	503±0.2		3.4±0.5		12.1±0.02	52.95 ±0.6	0.818±0.01	0.2	100.5±0.4	16.34±0.5	68±1.1

*In-vitro bioadhesion*

Sheep vaginal mucosa was used as a model membrane and SVF, pH 4.5 as moistening fluid for measurement of bioadhesive strength. A simple apparatus was devised to measure the minimum detachment force. Freshly obtained sheep Vaginal mucosal membrane was adhered to a piece of glass which was fixed on a plank and the plank was assembled with a little crown block. After hydrating the membrane with 20 µL of SVF the tablet was brought into contact with the membrane by applying 200 g for 2 min. After initial contact the tablet was encircled by a firm plastic ring which fastened a light plastic beaker through the crown block. Then water was dropped into the beaker at a speed of 1.5 mL/min until the tablet and membrane were pulled apart by the gravity of water. The beaker containing water was weighed and the minimum detachment force was calculated accordingly (19). 

*In-vitro* *drug release studies*

Tablet drug release was evaluated using a modified standard basket apparatus. A tablet side was wetted with 50 µL of simulated vaginal fluid and fixed to the bottom flat end of the stirring rod instead of the basket fixture. After 2 min, the vessel was filled with simulated vaginal fluid at 37 °C and stirred at 100 rpm speed. Samples (4 mL) were collected at predetermined time intervals and replaced with an equal volume of simulated vaginal fluid. Curcumin concentration in each sample was determined at 417 nm using UV Visible spectrometer (Shimadzu, 1800, Japan) (20). 

*In-vitro* *Antifungal Activity*

The antifungal activity of developed CBMV tablet was tested by disc diffusion method or cup plate method against candidia albicans j1023 (obtained from JSS Medical college). CBMV tablet was sterilized by autoclaving for 30 min at 120 °C and was placed in cultured agar plates. The plates are incubated for 2 days at 37 °C in an incubation chamber maintaining with 5% CO_2_ flow and the inhibition zone was then measured. Same procedure was followed for placebo tablet (tablet without drug) which acts as control in order to compare the antifungal activity with that of CBMV tablet.

*In-vivo X-ray studies*

The *in-vivo* X-ray studies were approved by the Institutional Animal Ethics Committee of JSS College of Pharmacy (Mysore, Karnataka, India). The study was performed on a healthy female rabbit, weighing between 1.5 and 2 kg. The optimized F3 formulation was modified by adding 20 mg of X-ray grade barium sulfate (20 mg curcumin was replaced). The prepared tablet was placed in the vaginal cavity of healthy rabbit. The rabbit was exposed to X-ray examinations and photographs were taken at 12 h after administration of the tablet.

*Kinetic analysis*

Drug release from curcumin monolithic vaginal tablets may be described by the power law expression and is defined by the following equation:

M_t_/M_∞_ = K_1_t_n _

where M_t_ is the amount of drug released at time t, M_∞_ is the overall amount of drug released, K_1_ is the release constant; n is the release or diffusional exponent and M_t_/M_∞_ is the cumulative drug concentration released at time t (or fractional drug release).

The release exponent (n) value was used for interpretation of the release mechanism from the tablets. The dissolution data were modeled by using PCP disso v2.01 (Bharathi Vidhyapeeth,

Deemed University, Pune, Maharashtra, India). 

## Results and Discussion

*Tablet characterization:*

The physicochemical properties of tablets are summarized in Table 2. Thickness of the tablet is also an important factor influencing drug release from monolithic tablet. As the tablet has to be inserted into the vaginal cavity, thickness of the tablet was given special care and measured. It was found that as the HPMC content is constant in the first six formulations (F1-F6) there was no change in the thickness of the tablet even though there is change in the concentration of other polymers like xanthan gum and guar gum. Formulation F8 showed lowest thickness compared to other formulations because of the absence of HPMC and thickness found to increase as the concentration of xanthan gum increased in F7 and F9 and in formulation F10 again the thickness increased due to presence of HPMC. Finally it can be concluded that all the formulations have the optimum thickness suitable to be inserted into the vaginal cavity. 

Hardness of the tablet is directly proportional to the force applied to compress a tablet. If the hardness exceeds 6 kg it influences the drug release from the monolithic tablet. In order to resist mechanical stress prepared tablet must show optimum compactness and hardness. Mean hardness of the prepared tablets ranged from 4.9-5.5 kg. There was no change in the hardness of tablets in formulation F1-F6. All the tablet formulations showed a higher strength which can resist mechanical stress. 

Mean tablet weight for all the prepared formulation was in the range of 499-504 mg. No batch varied by more than 5% from the tablet weight indicating consistency in tablet formulation and production. Concerning the uniformity of drug content, all of the formulations were acceptable since drug content of the tablet was 98.6-102% indicating uniform mixing of the tablet formulation. All the prepared tablets showed good compactness and friability less than 1%.

*FT-IR and DSC*

**Figure 1:**
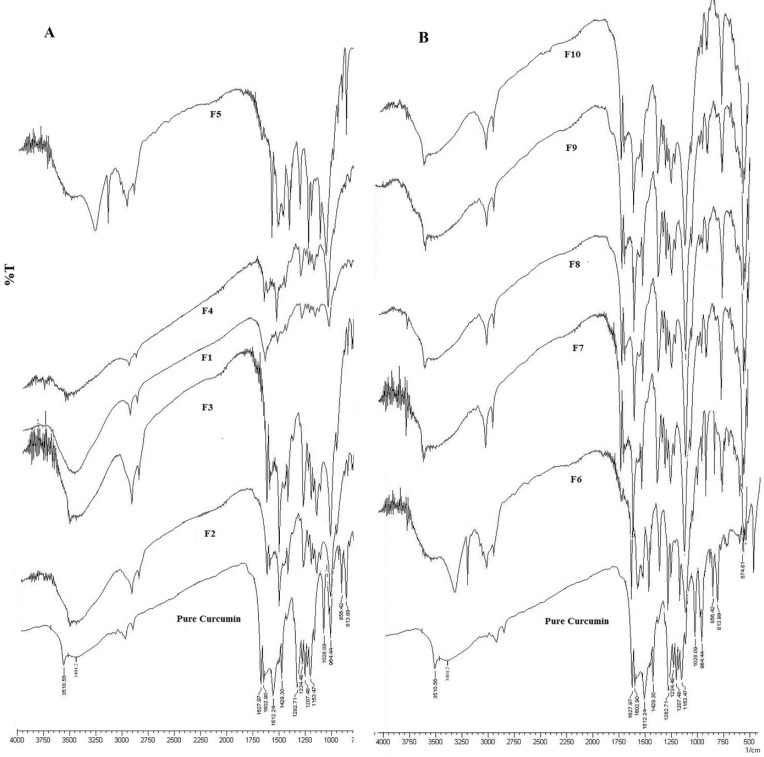
IR Spectra of pure curcumin and formulation (A) F1-F5 and (B) F6-F10.

The IR spectra of pure curcumin and formulations of curcumin are depicted in Figure 1. The IR spectra of curcumin and formulations were found to be identical. Pure curcumin showed stretching vibration of C = O (1640-1700 cm^-1^) and -OH (3400 cm^-1^) group appeared at wave number 1627.9 and 3404cm^-1^ respectively whereas ether group (–C-O-) was observed at 1026.7 cm^-1^, C = C aromatic stretching frequency was observed at 1429.3 cm^-1^, C = C olefinic stretching frequency was observed at 1512.2 cm^-1^ and a significant band was observed at 813.9 cm^-1^ being assigned to the C-H stretch.

The above mentioned characteristic peaks were also present in all the formulations F1- F10 without any changes in their positions indicating no chemical interactions between the curcumin and polymer used to formulate a tablet and compression of the curcumin in a tablet form using polymers like HPMC, xanthan gum, guar gum.

The results obtained by IR spectra indicating no chemical interaction between the drug and components of the formulations were further conformed by investigating using DSC analysis.

**Figure 2.**
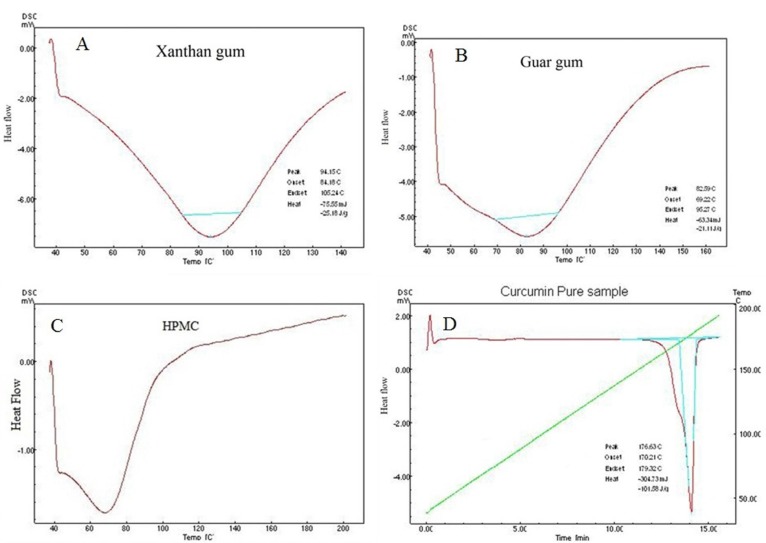
DSC analysis of pure polymers and pure curcumin

**Figure 3.**
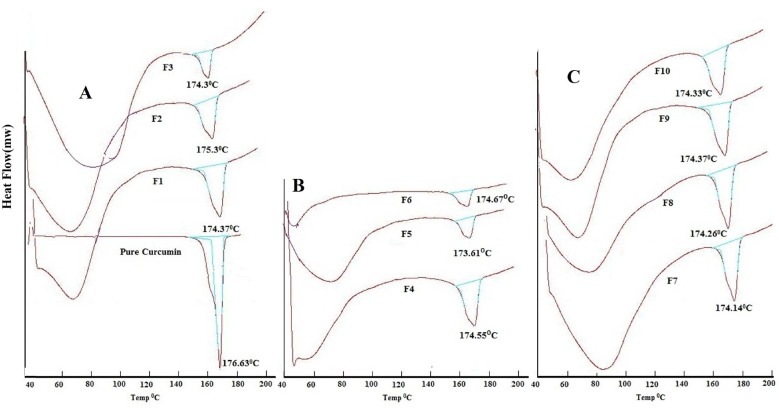
DSC analysis of (A) pure curcumin and formulation F1-F3, (B) F4-F6, (C) F7-F10

Analysis of pure curcumin resulted in gradual enthalpy change producing a linear sharp endothermic peak at temperature 176.63^o^C as shown in Figure 2D. DSC thermograms of polymers used are shown in Figure 2. Linearity and sharpness of the endothermic peak indicates the purity of the compound. DSC thermograms of the formulations F1-F10 failed to give the linear and sharp endothermic peak due to presence of polymers in the formulations which behaves as an impurity to the curcumin and hence resulted in a broad asymmetric melting peak at temperature varying from 173.6-175.38^o^C as shown in Figure 3. The melting point of endothermic peak of formulations was well within the acceptable limit indicating absence of drug and polymer interactions. 

*Swelling studies:*

Bioadhesive polymers used in the CBMV tablet swells in the presence of water; hence it is necessary to perform hydration studies in order to know the hydration capabilities. All tablets swelled after only 15 min. Initially, sharp ends of the tablet was getting smoothen to leave a gel layer on the tablet, gradually water penetration inside the tablet was making tablet to change its size and integrity. All tablets showed high hydration percentage. All tablets were found to retain their integrity even after 24 h. Formulations containing HPMC polymer (F1-F6) showed erosion of the tablet during swelling studies, but with formulations like F7, F8, F9 there was no erosion of the tablet which might be due to absence of HPMC. In formulation F7-F9 there was formation of a distinct gel layer on the surface of the tablet. Formulation F10 showed erosion of the tablet but to a lesser extent when compared to the formulation F1-F6. 

Combination of xanthan gum and guar gum resulted in a tablet which did not give significant erosion rate hence this combination will not be suitable for the release of curcumin.

*Bioadhesion*

**Figure 4:**
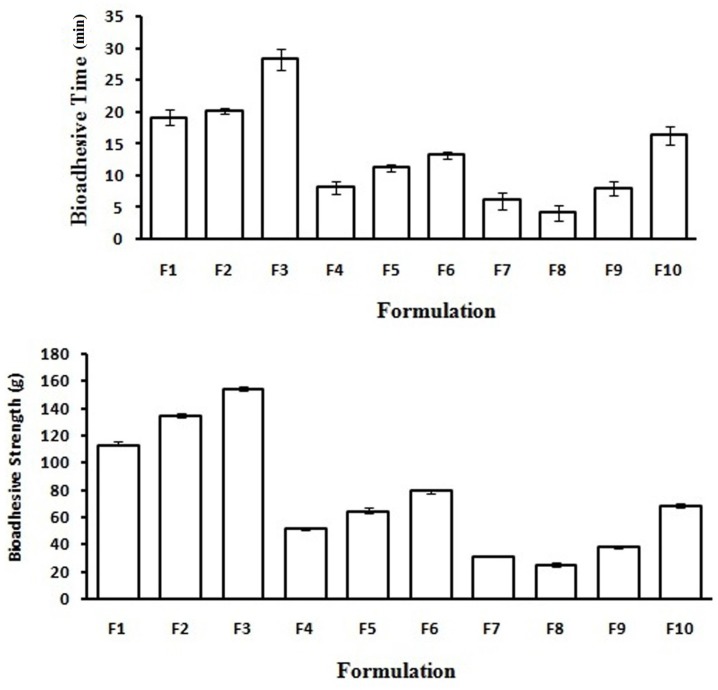
Comparison of bioadhesive strength and Ex-vivo mucoadhesive time of CBMV tablets.

Bioadhesion is the phenomenon between two materials which are held together for extended period of times by interfacial forces. It is generally referred to as bioadhesion when interaction occurs between polymer and epithelial surface. As discussed in the swelling studies about bioadhesive power of formulation which decreases as the hydration level increases because a large number of polymer binding sites are involved in bonds with water molecules, reducing thus the number of groups effectively available to interact with mucin chains. To achieve a release of the drug for prolonged period of time, formulation must adhere to mucosal surface. When a CBMV tablet is placed on sheep vaginal mucosa, wetting of the tablet leads to swelling of bioadhesive polymers at the interface which initiates intimate molecular contact at the interface between the polymer and the vaginal mucus layer. Formulation containing HPMC and xanthan gum exhibited better bioadhesion compared to other formulations which may be due to formation of secondary chemical bonds between the polymer chains and mucin molecules, F3 showed highest bioadhesion among all formulations. Formulations made with guar gum showed least bioadhesion which might be because of weak adhesive bonding across the interface. Comparison of bioadhesive strength and *ex-vivo* mucoadhesive time of curcumin tablet formulations is shown in Figure 4.

*In-vitro release studies:*

The ability of bioadhesive vaginal tablets of curcumin to remain intact in the physiological environment of vagina was assessed by conducting *In-vitro* drug release studies. And also the release rate of the drug from prepared controlled release formulation was determined.

**Figure 5:**
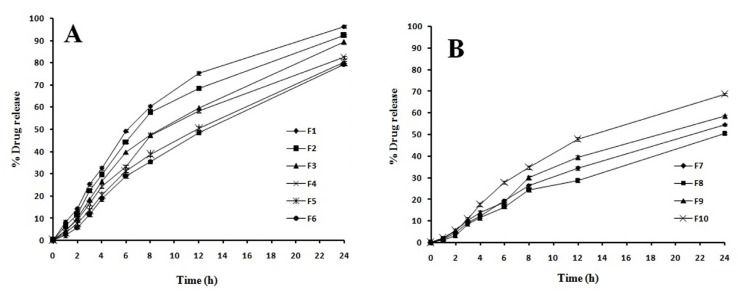
Effect of polymer type and ratio on the release of curcumin from CBMV tablets

As curcumin is a poor water soluble drug the release of the drug from tablet follows matrix erosion mechanism. Figure 5 shows the drug release profiles of the tablet formulations (F1-F10). It was observed from the data obtained that after 12 h, the release of drug was in the rank order F1 (96.33%) > F2 (92.22%) > F3 (89.22%) > F4 (82.33%) > F5 (80.22%) > F6 (79.22%) > F10 (68.78%) > F9 (58.43%) > F7 (54.67%) > F8 (50.44%). Use of HPMC in a matrix tablet as the only polymer will result in a very slow release of drug due to the buildup of an excessively viscous gel which is very resistant to water penetration and erosion. HPMC based systems has an initial burst effect in order to overcome this problem, therefore HPMC was mixed with xanthan gum and guar gum. A constant amount of HPMC was used in the formulation from F1-F6 and the concentration of xanthan gum and guar gum was a significant factor in the rate of drug release.

In formulation F1-F3 as the concentration of xanthan gum increases it reduces the drug release. Similar release pattern was observed with F4-F6; increase in guar gum concentration resulted in decreased drug release. There was no initial burst effect in all the formulations which may be due to rapid hydration and gelation of the polymers in the tablets. Previous studies reported that Fickian diffusion through the hydrated xanthan gum is not the only mechanism accounting for drug release but also swelling and erosion occurs concurrently resulting in moving frontier condition which continuously modifies the diffusivity of the drug. Xanthan gum as a hydrophilic polymer is capable of slowing drug release. Its effectiveness is due to its high swelling and solvent penetration rate coupled with its moderate erosion. Hence formulation F1-F3 showed higher erosion compare to other formulations as it has HPMC and Xanthan gum as a polymer.

Formuation F4-F6 showed a highly viscous gel around the tablet. Since the erosion rate of swollen gel is slow these formulations showed less release. The release of curcumin in formulation F7-F9 (50.44-58.43%) was very less compared to other formulations as there was lack of sufficient erosion of the tablet which is because of the absence of HPMC. In these formulations presence of xanthan gum showed high degree of swelling due to water uptake and small degree of erosion due to polymer relaxation. Hence it was observed from the study that combination of xanthan gum and guar gum alone without HPMC is not suitable for Curcumin as it releases by erosion mechanism. This combination of polymer is very much suitable for preparing matrix tablet for a water soluble drug which releases by diffusion mechanism. It was found that F1 & F2 formulations containing the combination of HPMC and Xanthan gum have better *in-vitro* release 96.36 ± 13 & 92.24 ± 31% respectively at 12 h release. 

The calculated parameters for interpretation of the release mechanism are given in Table 3. n-values of all formulations were found to be around 0.5, which indicates a drug release following the Fickian diffusion mechanism.

**Table 3:** Kinetic constants (k), release exponents (n) and determination coefﬁcients (r2) following linear regression of dissolution data of curcumin vaginal tablets

**Formulation**	**Zero order**			**Matrix**			**Korsmeyer-Peppas**		
	K(%hr^-1^)	r^2^	n	K(hr^-1^)	r^2^	n	K(hr^-1^)	r^2^	n
F1	29.09	0.984	0.57	27.76	0.9980	0.56	26.93	0.976	0.59
F2	26.93	0.9880	0.59	29.09	0.996	0.57	29.09	0.9780	0.57
F3	28.09	0.976	0.52	28.09	0.996	0.52	25.00	0.977	0.59
F4	27.76	0.977	0.56	25.00	0.984	0.59	32.24	0.981	0.60
F5	29.09	0.971	0.57	32.24	0.9980	0.60	28.09	0.976	0.52
F6	25.00	0.971	0.59	32.24	0.997	0.60	29.09	0.9780	0.57
F7	26.93	0.976	0.59	26.93	0.996	0.59	27.76	0.974	0.56
F8	32.24	0.977	0.60	25.00	0.991	0.59	25.00	0.977	0.59
F9	27.76	0.9780	0.56	29.09	0.984	0.57	27.76	0.974	0.56
F10	28.09	0.976	0.52	28.09	0.996	0.52	26.93	0.976	0.59

*In-vitro* *Antifungal Activity*

Curcumin showed antifungal activity against candida strains with minimum inhibitory concentrations (MIC) varying from 250-2000 µg/mL. Curcumin has shown more susceptibility to *Candida albicans* among the candida species studied. It was reported that curcumin is 2.5 fold more potent than fluconazole at inhibiting the adhesion of *C. albicans* to buccal epithelial cells (5). Curcumin shows its antifungal activity by generation of reactive oxygen species and triggers an early apoptosis in *Candida albicans* cells and alteration of membrane associated properties of ATPase activity, ergosterol biosynthesis and protein secretion. 

The antifungal activity of CBMV tablet and placebo tablet was evaluated by cup-plate method. The results were found encouraging. The placebo tablet has not shown any zone of inhibition. The zone of inhibition was found with the CBMV tablet. Antifungal study with Sabourad Culture shows that the CBMV tablet was capable to control the growth of *C. albicans *for more than 24 h. Zone of inhibition of CBMV tablet was found to be 19.5 ± 0.48 mm.

*In-vivo* X-ray studies

**Figure 6.**
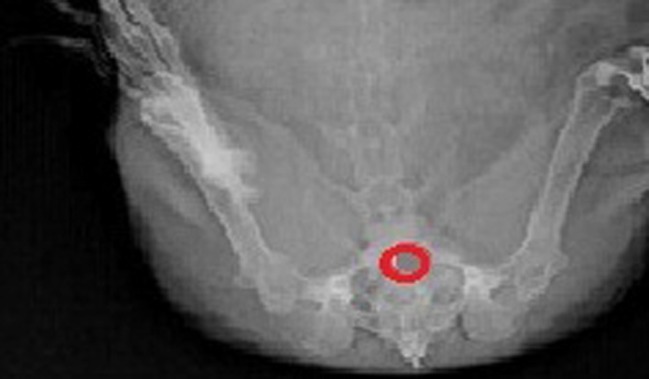
X-ray radiographic images of vaginal cavity at 8^th^ h after administration of BaSO_4_-loaded optimized F3 Vaginal tablet in female rabbit

After administration of optimized tablet formulation F3 having barium sulfate, the duration of the tablet in vaginal cavity was observed by radiogram shown in Figure 6. Tablet adheres to vaginal mucosa up to 12 h and swelling was observed after 1 h, after 8^th ^h the size of the tablet was slowly getting reduced.

## Conclusion

An herbal approach of using curcumin as an antifungal agent to treat vaginal candidiasis by formulating curcumin in a bioadhesive monolithic tablet was successful. The prepared curcumin bioadhesive vaginal tablet was effective against candida albicans. The burst effect of HPMC based systems was minimized by interaction of HPMC and xanthan gum which reported to form a gel on the surface of tablet. Combination of guar gum and HPMC yielded relatively lower bioadhesion under experimental condition and insufficient drug release due to lowest erosion rate. Hence, the use of guar gum is not a good bioadhesive polymer to use in vaginal tablets and release of curcumin. The prepared tablets are expected to offer a patient compliant once-a-day vaginal bioadhesive formulation for sustained local effect of curcumin in vaginal candidiasis. Among all formulations F3 was suitable to administer into the vagina as it was found to have good mucoadhesion and showed optimum thickness suitable to be inserted into the vaginal cavity. The combination of all results showed that curcumin bioadhesive tablet prepare using HPMC and xanthan gum exhibited interesting swelling, mucoadhesive, and release properties with a very good antifungal activity making them promising formulations for the vaginal administration of curcumin which is an herbal approach for treating vaginal candidiasis. Further various other suitable vaginal formulations of curcumin can be developed due to the antifungal activity of curcumin. The results of this preparation have to be tested *in-vivo*, in real life.
